# Survey of overwintering *Halyomorpha halys* (Hemiptera: Pentatomidae) in ports of export and natural landscapes surrounding the ports in Republic of Korea

**DOI:** 10.1371/journal.pone.0270532

**Published:** 2022-08-26

**Authors:** Hyunsung Song, Minhyung Jung, Seoyul Hwang, Jiseok Kim, Donghun Kim, Doo-Hyung Lee

**Affiliations:** 1 Department of Life Sciences, Gachon University, Seongnam-si, Gyeonggi-do, South Korea; 2 Department of Vector Entomology, Kyungpook National University, Sangju, Gyeongsangbok-do, South Korea; 3 Department of Plant Protection and Quarantine, Graduate School of Plant Protection and Quarantine, Kyungpook National University, Daegu, South Korea; University of Carthage, TUNISIA

## Abstract

*Halyomorpha halys* (Hemiptera: Pentatomidae), an important agricultural and nuisance pest, is highly invasive with peculiar hiding behavior in human-made structures for overwintering. To evaluate the contamination risk of overwintering *H*. *halys* in non-agricultural export goods, we conducted a two-year field survey in Republic of Korea to locate overwintering *H*. *halys* in two major ports of export, Ulsan and Pyeongtaek ports, and monitored both active and overwintering *H*. *halys* population levels with varying distances from the ports ranging from 1 km to 48 km. First, we deployed wooden shelters in the two ports to catch dispersing *H*. *halys* for overwintering and conducted visual inspections for human-made structures in the ports to locate overwintering *H*. *halys*. In addition, we sampled dead trees to find overwintering *H*. *halys* in wooded areas. Second, we monitored active *H*. *halys* populations using pheromone traps with varying distances from the ports. From the survey of overwintering populations, no *H*. *halys* was collected from wooden shelters deployed in the two ports. However, we found four adults overwintering in human-made structures in Pyeongtaek port in the first year of survey. One dead adult was also found from a dead tree located in a wooded area adjacent to Pyeongtaek port in the second year. For active populations, results of pheromone trapping indicated that *H*. *halys* populations were present during autumn dispersal period not only in agricultural areas, but also in wooded areas adjacent to the two ports. This study reports for the first time that overwintering *H*. *halys* were found from the inside the port of export in its native areas with a low density. The results were discussed for evaluating contamination risk of overwintering *H*. *halys* in export goods shipping from the Republic of Korea.

## Introduction

Although a small fraction of introduced species can successfully establish in new areas [1–3], the number of invasive species has dramatically increased with the growth of world trade since 1900s, especially via accidental introductions [4]. *Halyomorpha halys* (Stål) (Hemiptera: Pentatomidae), a brown marmorated stink bug, is a highly invasive pest originating from East Asia, which has rapidly expanded its global distribution [5]. Since the first detection of *H*. *halys* in Pennsylvania, U.S.A. in 1996 [6], invasion of *H*. *halys* was reported in 35 countries as of April 2021 [7]. This rapid geographical expansion of *H*. *halys* is thought to have resulted from hitchhiking in shipping goods with its overwintering behavior and successful establishment with a wide range of host plants [8], strong flight capacity [9, 10], high reproductive rate [11], and low suppression by indigenous natural enemies [12]. Moreover, human-assisted movement of *H*. *halys* has resulted in a rapid and erratic establishment of this pest compared to natural spread of other invasive insects in the past [13].

*Halyomorpha halys* causes serious problems during both active and overwintering seasons, especially in introduced areas. During the active season, both nymphs and adults attack economically important crops such as apples, peaches, pears, and grapes with their wide host range including more than 170 plant species [5, 8]. Indeed, it was estimated that *H*. *halys* caused more than €500 million losses to fruit crops in Italy in 2019 [14] and US$ 52.7–68.6 million losses to hazelnut in Georgia in 2016 [15]. During the overwintering season, *H*. *halys* adults cause serious nuisance problems by aggregating in artificial structures such as buildings, homes, and sheds [16–18]. Overwintering of *H*. *halys* begins with dispersing to overwintering sites in late September, and this dispersal increases by October [8, 19]. During this period, very high numbers of *H*. *halys* adults often aggregate on the exterior of artificial structures [16, 17], and they crawl and settle in dry and tight gaps at dark locations of the structures such as attics [17, 20, 21].

This characteristic overwintering behavior of *H*. *halys* provides opportunity for this insect to rapidly expand its geographical range via hitchhiking in shipped goods [6, 21, 22]. For example, genetic diversity analysis suggests that *H*. *halys* in Greece have established through multiple and/or large introductions of the insects from Asia [23]. *Halyomorpha halys* adults are thought to be introduced and have established in the U.S., Canada, Italy, and Romania via international commerce [6, 24–26]. Although the establishment of *H*. *halys* in Oceanian countries including Australia and New Zealand has not been reported, interception of *H*. *halys* at their borders has been repeatedly reported [27, 28]. In Australia, for example, live *H*. *halys* adults have been intercepted from goods such as vehicles, machinery, and their parts imported from northern hemisphere arriving between September and May [21]. Recently, several cargo vessels carrying thousands of cars from Republic of Korea were refused entry to Australia due to the detection of overwintering *H*. *halys* in either vessels or cars [29]. This quarantine issue caused substantial economic loss due to required treatment and shipping delay.

Under these circumstances, understanding the risk of *H*. *halys* contamination in non-agricultural export goods during overwintering dispersal period is essential to mitigate the contamination by this pest, thereby preventing it from invading new countries. That is, this information may be crucial for countries at invasion risk to design prevention strategies and for native countries to develop management programs. Therefore, in this study, we conducted a two-year field survey to locate overwintering *H*. *halys* in two major ports of export in Republic of Korea and monitor both active and overwintering *H*. *halys* population levels with varying distances from the two ports. Based on results of field survey, we discussed risk levels of the two ports of export to harbor overwintering *H*. *halys* and their contamination risk when exporting goods.

## Materials and methods

### Ethics statement

Permission was obtained through Animal and Plant Quarantine Agency (APQA) for entry to Ulsan and Pyeongtaek ports to survey overwintering *H*. *halys* in port areas and facilities.

### Overwintering population

Overwintering *H*. *halys* populations were surveyed in two regions: Ulsan and Pyeongtaek, Republic of Korea (Table 1). The survey was conducted from September 2019 to February 2020 (1st year) and from September 2020 to February 2021 (2nd year).

**Table 1:** Information of sampling sites for surveying active and overwintering *Halyomorpha halys* populations in Ulsan and Pyeongtaek regions.

Region	Sampling site	Target population	Sampling year	Sampling period	Distance from port (km)	Crop	Area size (m^2^)	GPS coordinates
Ulsan	Port	Overwintering	1st & 2nd	Sep–Feb	-	-	250,217	35°31’17.63”N 129°23’32.67”E
	Wooded area 1	Active / Overwintering	1st / 1st & 2nd	Oct–Nov / Feb	0.8	-	4,080	35°31’08.55”N 129°23’57.68”E
	Wooded area 2	Overwintering	2nd	Feb	0.9	-	1,600	35°31’38.54”N 129°24’00.20”E
	Agricultural area 1	Active	1st	Oct–Nov	11.8	Pear	19,600	35°31’45.36”N 129°15’46.58”E
	Agricultural area 2	Active	1st	Oct–Nov	12.0	Apple	18,500	35°36’44.35”N 129°19’16.73”E
	Wooded area 3	Overwintering	2nd	Feb	16.3	-	6,810	35°29’58.92”N 129°12’50.71”E
	Agricultural area 3	Active	1st	Oct–Nov	47.8	Apple	3,242	35°34’37.07”N 128°52’09.40”E
Pyeongtaek	Port	Overwintering	1st & 2nd	Sep–Jan	-	-	209,359	36°58’35.41”N 126°50’09.35”E
	Wooded area 1	Active / Overwintering	1st / 1st & 2nd	Oct–Dec / Feb	1.8	-	12,420	36°58’12.09”N 126°51’11.02”E
	Agricultural area 1	Active	1st	Oct–Dec	12.5	Apple	7,300	36°56’57.97”N 126°58’06.35”E
	Agricultural area 2	Active	1st	Oct–Dec	13.0	Pear	34,500	37°02’50.03”N 126°56’51.30”E
	Wooded area 2	Overwintering	2nd	Jan	27.9	-	10,610	37°01’10.97”N 127°08’24.55”E
	Agricultural area 3	Active	1st	Sep–Dec	38.8	Pear	21,800	37°01’46.84”N 127°15’44.35”E

**Fig 1:**
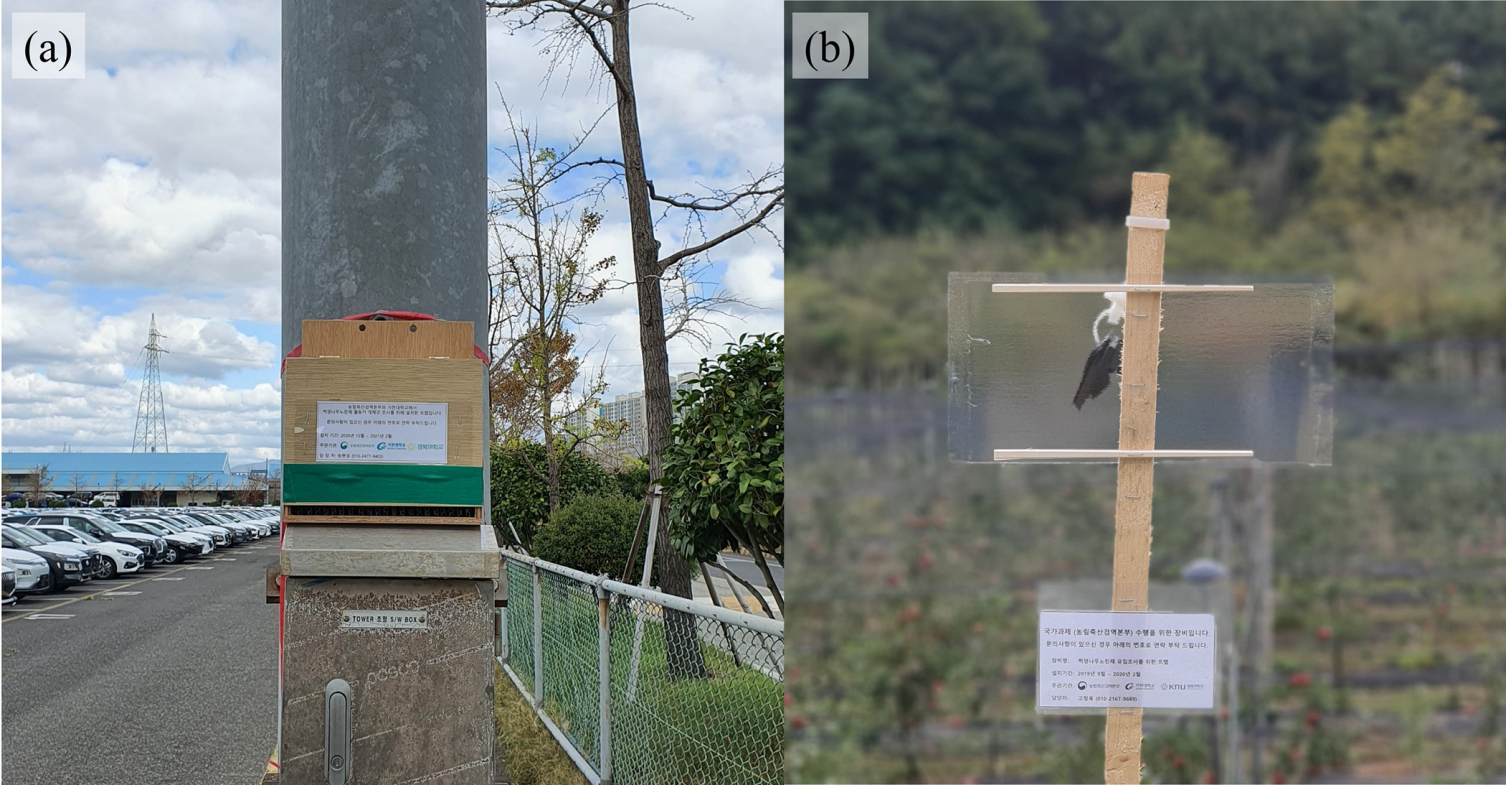
Representative photos of (a) overwintering shelter and (b) clear sticky pheromone trap, to detect overwintering and active *Halyomorpha halys*, respectively.

First, to detect *H*. *halys* dispersing to their overwintering sites, wooden shelters (W × D × H: 24 × 20 × 20 cm) were deployed onto artificial structures in the two ports (Fig 1A). These shelters were originally developed by Bergh *et al*. [30] and modified for use in this study. The structure does not include attractant for *H*. *halys*, but is designed to provide tight and dry crevices inside the shelter serving as favorable microstructures for overwintering *H*. *halys*. The shelters were deployed on the surface of larger structures such as buildings. They were deployed in September or October because most adult *H*. *halys* are known to disperse to overwintering sites during September and October [8, 19]. In the first year, 20 shelters were deployed in Ulsan port; in the second year, 20 shelters each were deployed in Ulsan and Pyeongtaek ports. They were set up at ca. 0.5–2.5 m from the ground to avoid moisture [20]. The shelters were collected and brought to the laboratory in January or February for inspection.

Second, export cars waiting to be shipped to New Zealand or Australia in these two ports were inspected in September to locate overwintering *H*. *halys* in cars. Forty-seven cars in Ulsan port were examined on September 27, 2019 and 55 cars in Pyeongtaek port were inspected on September 18, 2020. Based on the settling behavior of overwintering *H*. *halys* [20, 31], inspectors made effort to find and check tight and dry gaps in the cars. Gaps were found mainly from spaces beneath vinyl wrapped on car exteriors, between doors and rubber packing, between tires and wheels, or among compartments in the engine room. Average inspection time for each car was about 2.4–3.0 minutes.

**Fig 2:**
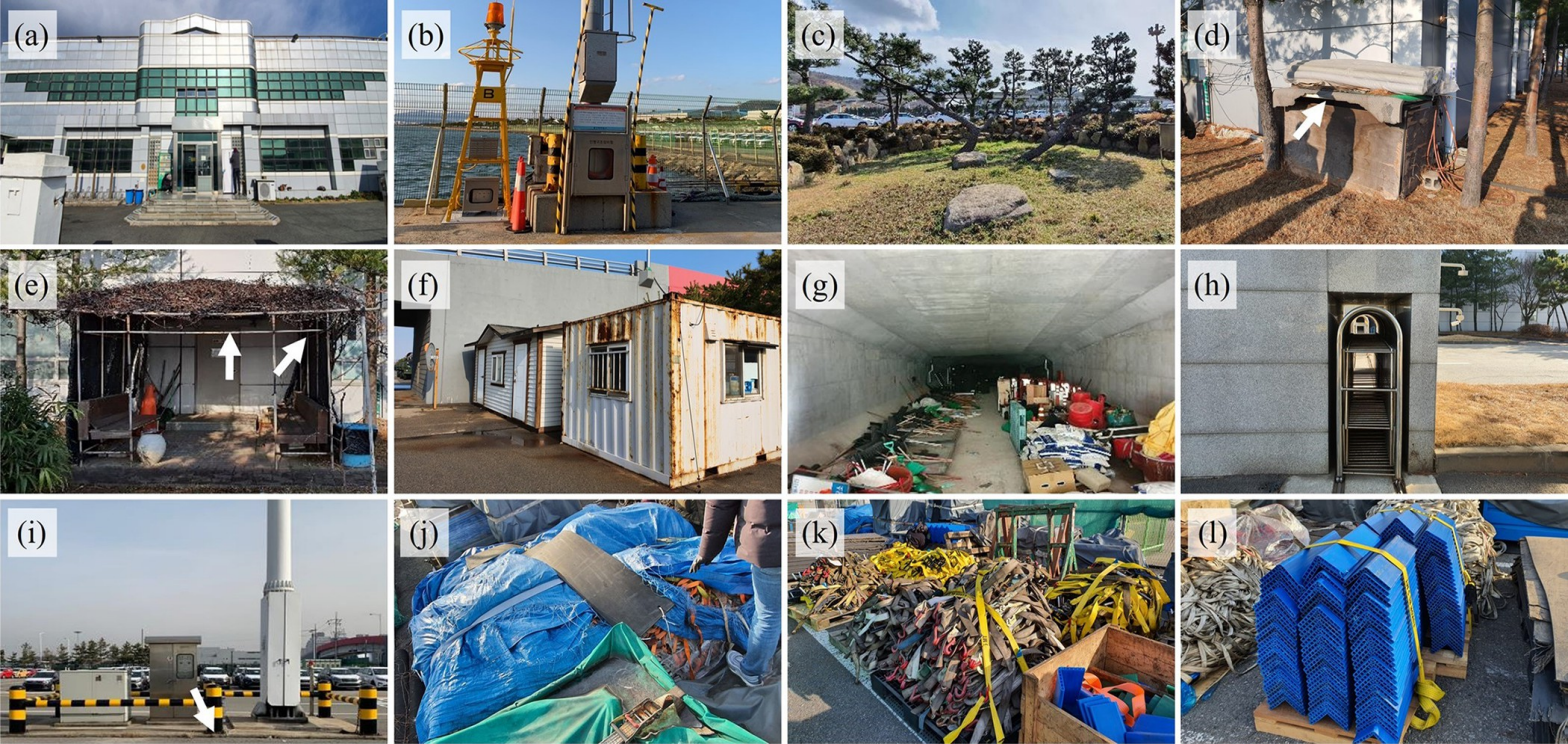
Representative photos of structures inspected to detect overwintering *Halyomorpha halys* in Ulsan and Pyeongtaek ports, Republic of Korea. (a) Building exterior (U1 in Table 2), (b) CCTV tower (U38), and (c) Stone boundary & rocky crevice (U42-45) in Ulsan port, and (d) Building appendix (P3), (e) Rest area (P4), (f) Container exterior (P7), (g) Warehouse (P12-16), (h) Gate fence (P22), (i) Light tower (P33), (j) Tarpaulin (P42), (k) Lashing (P51), and (l) Stacked plastic panels (P55) in Pyeongtaek port. White arrows indicate locations of overwintering *H*. *halys* found by visual inspection.

Third, structures in the two ports were visually inspected to detect overwintering *H*. *halys* adults in these ports in January or February (Fig 2; Table 2). Similar to visual inspection for cars, tight and dry gaps of structures were mainly inspected as potential overwintering habitats of *H*. *halys*. Because these structures substantially varied in size and complexity, sampling efforts for inspected structures were standardized as follows. Prior to inspection, six inspectors evaluated structures to score the structures with regard to their likelihoods to be used as overwintering habitats by *H*. *halys*. Each structure was evaluated with a score scale of 1, 2, 3, 4, and 5, with higher scores for structures having more and larger micro-structures known to be favored by *H*. *halys* as their overwintering habitats (e.g., tight and dry gaps between wood panels) (Fig 2; Table 2) [15, 20, 31]. For example, stacked cardboard boxes and stored goods covered by tarpaulin were ranked higher (Figs 1J and 2D; P3, P42 in Table 2), whereas light towers were ranked lower because the towers had very few tight and dry gaps (Fig 2I; P33 in Table 2). Sampling intensity was determined by considering both the likelihood evaluation score and the size of the structure (Table 2). In the first year of the survey, 50 and 57 structures in Ulsan and Pyeongtaek ports, respectively, were inspected. In the second year, 35 structures were inspected for each port.

**Table 2:** Information of structures inspected to detect overwintering *Halyomorpha halys* in Ulsan and Pyeongtaek ports, Republic of Korea (see Fig 2 for representative photos of inspected structures).

Structure ID^a^	Structure	Sampling year	Mean likelihood^b^	Structure size (m^2^)	Sampling intensity (min)^c^	*H*. *halys* detected
U1	Building exterior	1st / 2nd	1.57	172.26	54.30 / 21.10	0 / 0
U2	Building roof	1st / 2nd	2.29 / 3.00	330.00	20.00 / 19.20	0 / 0
U3	Rest area 1	1st / 2nd	2.57	30.00	22.00 / 10.00	0 / 0
U4	Rest area 2	1st / 2nd	2.43	30.00	20.00 / 10.00	0 / 0
U5	Rest area 3	1st / 2nd	2.00	6.82	20.50 / 8.00	0 / 0
U6	Building appendix 1	1st / 2nd	3.71	5.96	10.00 / 5.00	0 / 0
U7	Building appendix 2	1st / 2nd	1.29	1.86	5.00 / 5.00	0 / 0
U8	Building appendix 3	1st / 2nd	2.57 / 2.00	1.92	6.00 / 5.00	0 / 0
U9	Cement fence	1st / 2nd	2.29	11.35	21.00 / 7.68	0 / 0
U10	Light tower 1	1st / 2nd	1.57	9.24	3.00 / 2.08	0 / 0
U11	Light tower 2	1st / 2nd	1.57	9.24	3.00 / 2.17	0 / 0
U12	Light tower 3	1st / 2nd	1.57	11.44	5.00 / 2.13	0 / 0
U13	Light tower 4	1st	1.57	8.61	3.00	0
U14	Light tower 5	1st / 2nd	1.57	8.61	3.00 / 2.28	0 / 0
U15	Light tower 6	1st / 2nd	1.57	8.61	3.00 / 2.08	0 / 0
U16	Light tower 7	1st / 2nd	1.57	8.61	3.00 / 2.17	0 / 0
U17	Light tower 8	1st	1.57	10.71	3.00	0
U18	Light tower 9	1st	1.14	6.76	2.00	0
U19	Light tower 10	1st	1.14	6.76	2.00	0
U20	Light tower 11	1st / 2nd	1.14	6.76	3.00 / 2.00	0 / 0
U21	Light tower 12	1st	1.14	6.76	2.00	0
U22	Light tower 13	1st	1.14	6.76	2.00	0
U23	Light tower 14	1st	1.14	6.76	2.00	0
U24	Light tower 15	1st / 2nd	1.14	6.76	2.00 / 2.00	0 / 0
U25	Light tower 16	1st	1.14	6.76	2.00	0
U26	Light tower 17	1st	1.14	6.76	2.00	0
U27	Light tower 18	1st / 2nd	1.14	6.76	2.00 / 2.00	0 / 0
U28	Light tower 19	1st	1.14	6.76	2.00	0
U29	Light tower 20	1st	1.14	6.76	2.00	0
U30	Light tower 21	1st / 2nd	1.14	6.76	2.00 / 2.00	0 / 0
U31	Light tower 22	1st / 2nd	1.14	6.76	2.00 / 2.00	0 / 0
U32	Light tower 23	1st	1.14	6.76	2.00	0
U33	Light tower 24	1st / 2nd	1.14	6.76	2.00 / 2.00	0 / 0
U34	Light tower 25	1st / 2nd	1.14	6.76	2.00 / 2.00	0 / 0
U35	Light tower 26	1st / 2nd	1.14	6.76	2.00 / 2.00	0 / 0
U36	Light tower 27	2nd	1.14	6.76	2.00	0
U37	Light tower 28	2nd	1.14	6.76	2.00	0
U38	CCTV tower 1	1st / 2nd	2.33	6.61	4.00 / 3.35	0 / 0
U39	CCTV tower 2	1st / 2nd	2.33	5.29	3.00 / 2.67	0 / 0
U40	Gas duct	1st / 2nd	1.00	253.00 / 10.00	10.00 / 7.00	0 / 0
U41	Metal frame	1st / 2nd	1.43	1.00	3.00 / 3.00	0 / 0
U42	Stone boundary	1st / 2nd	3.00	140.00	51.60 / 44.40	0 / 0
U43	Rocky crevice 1	1st / 2nd	2.29	6.20	2.00 / 1.00	0 / 0
U44	Rocky crevice 2	1st / 2nd	2.29	6.60	2.50 / 1.00	0 / 0
U45	Rocky crevice 3	1st / 2nd	2.29	6.60	1.00 / 1.00	0 / 0
U46	Rocky crevice 4	1st / 2nd	2.29	7.40	2.50 / 1.00	0 / 0
U47	Rocky crevice 5	1st / 2nd	2.29	6.00	1.78 / 1.00	0 / 0
U48	Tree base 1	1st	4.29	0.11	1.50	0
U49	Tree base 2	1st	4.29	0.06	1.00	0
U50	Tree base 3	1st	4.29	0.08	2.30	0
U51	Tree base 4	1st	4.29	0.23	1.50	0
U52	Leaf litter	1st	2.17	0.42	2.00	0
P1	Building 1 exterior	1st / 2nd	1.14	162.00	30.00 / 34.00	0 / 0
P2	Building 1 roof	1st / 2nd	1.57	450.00	20.00 / 20.00	0 / 0
P3	Building 1 appendix	1st / 2nd	3.57 / 1.80	1.50	30.00 / 5.00	1 / 0
P4	Rest area	1st / 2nd	2.43	120.00	20.00 / 20.17	2 / 0
P5	Building 2 exterior	1st / 2nd	1.60	270.00	50.00 / 31.32	0 / 0
P6	Building 2 roof	1st / 2nd	3.60	780.00	20.00 / 8.75	0 / 0
P7	Container 1 exterior	1st / 2nd	1.60	27.00 / 18.00	28.00 / 8.67	0 / 0
P8	Container 1 interior	1st	3.60	9.00	30.00	0
P9	Container 2 exterior	1st / 2nd	2.14	129.00	30.00 / 30.50	0 / 0
P10	Container 2 interior 1	1st	1.86	12.00	10.00	0
P11	Container 2 interior 2	1st	2.57	18.00	21.00	0
P12	Warehouse 1 section A	1st / 2nd	1.86	34.32	16.00 / 6.52	0 / 0
P13	Warehouse 1 section B	1st / 2nd	1.29	16.50	13.00 / 9.50	0 / 0
P14	Warehouse 1 section C	1st / 2nd	2.29	26.00	12.00 / 5.50	0 / 0
P15	Warehouse 1 section D	1st / 2nd	2.43	28.80	36.00 / 12.00	0 / 0
P16	Warehouse 1 section E	1st / 2nd	2.86	10.92	28.00 / 9.38	0 / 0
P17	Warehouse 2 section A	1st / 2nd	3.43	9.35	17.00 / 4.23	0 / 0
P18	Warehouse 2 section B	1st / 2nd	2.00	9.20	2.00 / 16.83	0 / 0
P19	Warehouse 2 section C	1st / 2nd	2.71	12.88	20.00 / 9.50	0 / 0
P20	Warehouse 2 section D	1st / 2nd	1.00	6.12	6.00 / 4.57	0 / 0
P21	Gate 1 office	1st / 2nd	1.57	68.40	4.00 / 5.00	0 / 0
P22	Gate 1 fence 1	1st / 2nd	1.00	12.60	3.00 / 3.00	0 / 0
P23	Gate 1 fence 2	1st / 2nd	1.00	12.60	3.25 / 3.00	0 / 0
P24	Gate 2 first floor	1st / 2nd	1.20	39.45	6.60 / 6.10	0 / 0
P25	Gate 2 second floor	1st / 2nd	1.40	21.80	14.00 / 10.00	0 / 0
P26	Waste cage	1st / 2nd	1.40	9.46	15.00 / 5.30	0 / 0
P27	Light tower 1	1st / 2nd	1.71	12.00	3.00 / 2.00	0 / 0
P28	Light tower 2	1st / 2nd	1.71	12.00	4.30 / 2.00	0 / 0
P29	Light tower 3	1st / 2nd	1.71	12.00	3.00 / 2.00	0 / 0
P30	Light tower 4	1st / 2nd	1.71	12.00	3.00 / 2.00	0 / 0
P31	Light tower 5	1st / 2nd	1.71	12.00	3.00 / 2.00	0 / 0
P32	Light tower 6	1st / 2nd	1.71	12.00	3.00 / 2.00	0 / 0
P33	Light tower 7	1st / 2nd	1.71	12.00	3.00 / 2.00	1 / 0
P34	Light tower 8	1st / 2nd	1.71	12.00	3.00 / 2.00	0 / 0
P35	Light tower 9	1st / 2nd	1.71	12.00	3.00 / 2.00	0 / 0
P36	Light tower 10	1st / 2nd	1.71	12.00	3.00 / 2.00	0 / 0
P37	Light tower 11	1st / 2nd	1.71	12.00	5.00 / 2.00	0 / 0
P38	Light tower 12	1st / 2nd	1.71	12.00	3.00 / 2.00	0 / 0
P39	Tarpaulin 1	1st	3.67	1.30	6.00	0
P40	Tarpaulin 2	1st	3.67	1.30	4.00	0
P41	Tarpaulin 3	1st	3.67	1.30	16.00	0
P42	Tarpaulin 4	1st	4.33	-	41.20	0
P43	Tarpaulin 5	1st	1.67	1.21	5.20	0
P44	Tarpaulin 6	1st	2.00	1.21	9.20	0
P45	Tarpaulin 7	1st	1.33	1.21	4.00	0
P46	Tarpaulin 8	1st	1.67	1.08	2.00	0
P47	Tarpaulin 9	1st	1.67	1.08	4.00	0
P48	Tarpaulin 10	1st	2.00	1.08	4.00	0
P49	Lashing 1	1st	2.00	1.30	2.30	0
P50	Lashing 2	1st	2.00	1.30	1.60	0
P51	Lashing 3	1st	2.00	1.30	3.00	0
P52	Lashing 4	1st	2.00	1.30	2.60	0
P53	Lashing 5	1st	2.00	1.30	3.50	0
P54	Lashing 6	1st	2.00	1.30	5.50	0
P55	Stacked plastic panel 1	1st	3.50	1.20	15.60	0
P56	Stacked plastic panel 2	1st	3.50	0.50	22.00	0
P57	Stacked plastic panel 3	1st	3.50	0.56	15.00	0

^*a*^Letters ‘U’ and ‘P’ indicate Ulsan and Pyeongtaek ports, respectively.

^*b*^Likelihood evaluation was made with a score of 1 to 5. Higher scores were assigned when structures had more and larger micro-structures known to be favored by *H*. *halys* as their overwintering habitats. Mean likelihood is the average score of six inspectors.

^*c*^Sampling intensity was calculated as the sum of inspection time (min) per investigator for each structure.

In addition to sampling inside the two ports, dead trees were inspected to find overwintering *H*. *halys* in wooded areas with varying distances from the two ports (Table 1). Dead trees were selected for inspection based on results of a previous study [32]. They were destructively sampled to locate overwintering *H*. *halys* adults beneath tree barks or in decomposing tissues [32]. Inspectors made efforts to find up to 30 dead trees at each sampling site. Each tree was sampled for 7.1 and 9.0 min on average in Ulsan and Pyeongtaek regions, respectively. In the Ulsan area, 27 trees were sampled in two wooded areas adjacent to the port (wooded areas 1 and 2) and 26 trees were sampled in one wooded area in the vicinity of an agricultural area (wooded area 3) in February 2021 (Table 1). In the Pyeongtaek area, 30 trees each were sampled in a wooded area adjacent to the port (wooded area 1) and in the vicinity of an agricultural area (wooded area 2) in January 2021 (Table 1).

### Active populations

Pheromone traps were deployed to monitor population density of active *H*. *halys* among the four monitoring sites with varying distances from each port (Fig 1B; Table 1). Active populations were monitored during early October through late November in Ulsan and mid-October through early December in Pyeongtaek in 2019. At each monitoring site, 10 pheromone traps were deployed with ca. 10-m distance away from each other. In agricultural areas, pheromone traps were deployed ca.10 m away from crop borders. These traps consisted of commercially-available pheromone lures (Green Agro Tech Inc., Kyungsan-si, Gyeongsangbuk-do, Korea) and double-sided clear sticky panels (15 × 30 cm) (AgBio Inc., Westminster, CO, USA) following the trap design used by Acebes-Doria *et al*. [33]. Two doses of lures were used in this study: 1) a high loading dose (20 mg of *H*. *halys* aggregation pheromone and 200 mg of methyl (2*E*,4*E*,6*Z*)-decatrienoate (MDT) pheromone synergist); and 2) a low loading dose (one fourth of the high dose). Low-dose traps were used for wooded areas adjacent to the ports (wooded area 1) due to concerns of attracting excessive *H*. *halys* to vicinities of these ports (Table 1). Sticky panels were replaced every two weeks, whereas lures were maintained throughout the eight-week monitoring period following the study of Acebes-Doria *et al*. [33]. These sticky panels were brought to the laboratory and checked for *H*. *halys*. Mean numbers of *H*. *halys* collected over two weeks per trap were compared among the four monitoring sites using ANOVA followed by Tukey’s HSD (JMP 12, SAS Institute Inc., NC, USA).

## Results

### Overwintering populations

No overwintering *H*. *halys* adults were found in wooden shelters deployed in either Ulsan or Pyeongtaek port throughout the two-year survey period. Likewise, no *H*. *halys* were found from visual inspection of cars waiting to be shipped in the two ports.

From visual inspection of human-made structures in the two ports, four overwintering *H*. *halys* adults were found in Pyeongtaek port in the first-year survey (Fig 2; Table 2). All overwintering adults were found alive and solitary in structures. The first overwintering *H*. *halys* was a female found in a gap created between cardboard boxes piled above a wooden frame (ca. 1 m above the ground) located next to a building wall facing southeast (Fig 2D; P3 in Table 2). The second and third *H*. *halys* were males found in a shade structure attached to the same building side from which the first individual was found. The second individual was found from a ca. 7-mm gap created between vinyl sheets and a bamboo frame supporting the shade established over a building entrance at ca. 2.4 m from the ground (Fig 2E; P4 in Table 2). The third individual was found from a ca. 4-mm gap created by a plastic panel placed on a cement eave above the building entrance at ca. 2.5 m above the ground (Fig 2E; P4 in Table 2). The last individual was a male found from a crevice ca. 12-mm in the ground made of cement under a light tower (Fig 2I; P33 in Table 2). The crevice was located on the southwestern side of the light tower.

One dead *H*. *halys* female was also found from a dead tree in the wooded area adjacent to Pyeongtaek port (Table 1). This adult was found beneath the bark of a standing dead coniferous tree with a diameter of 20-cm at breast height. The tree was located in a wooded area with both deciduous and coniferous tree species, ca. 20 m away from the boundary of a small vegetable garden.

### Active populations

**Fig 3:**
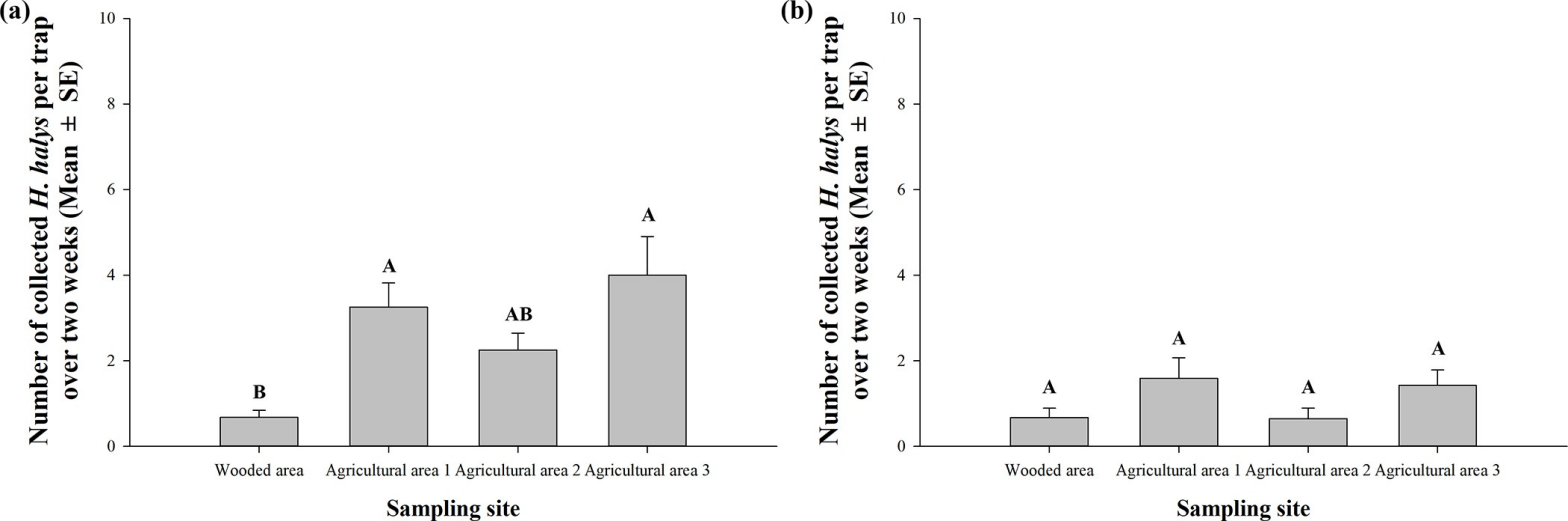
The number of *Halyomorpha halys* collected over two weeks per pheromone trap in early October through late November in Ulsan region (a) and mid-October through early December in Pyeongtaek region (b). Note that low dose of pheromone lures was used in wooded area (see Materials and Methods). Different letters indicate significant difference among sampling sites (*p* < 0.05).

*Halyomorpha halys* populations were collected using pheromone traps in four sampling sites surrounding each port between October and December in 2019 (Table 1). Overall, the number of *H*. *halys* collected in Ulsan region was 2.7 times greater than that in Pyeongtaek region; a total of 458 and 172 individuals were caught in Ulsan and Pyeongtaek regions, respectively. These collected individuals were adults except for two 5th instar nymphs collected in mid-October from Ulsan and late-October from Pyeongtaek. In Ulsan region, the number of *H*. *halys* collected in the wooded area was significantly lower than those collected from agricultural areas 1 and 3 (*F* = 5.3812, *df* = 3, 156, *p* = 0.0015) (Fig 3A). Sex ratio of the collected adults was 1.6:1.0 (F:M). On the other hand, in Pyeongtaek region, there was no significant difference in the number of *H*. *halys* collected among the four locations (*F* = 2.0846, *df* = 3, 155, *p* = 0.1045) (Fig 3B). Sex ratio of the collected adults was 1.4:1.0 (F:M).

## Discussion

Export ports are considered one of the main invasion pathways of *H*. *halys* because this pest often hides in shipping goods and vessels for overwintering during its autumn dispersal period [6]. Indeed, this invasive species is thought to be introduced into several countries during the overwintering season via international commerce [6, 24–26]. In Australia, live *H*. *halys* were intercepted from cargo vessels carrying shipping goods in September through May, with dead individuals intercepted throughout the year except for August [20]. In addition, live individuals have been intercepted from ports and warehouses where imported goods are stored in Australia and New Zealand [27, 34]. For these reasons, these countries have practiced rigorous quarantine measures to prevent *H*. *halys* invasion. Prior to entry to their ports, fumigation or heat practices are mandated by governments to export vessels, especially for those departing from *H*. *halys* high risk countries such as Italy or the U.S. from September to April [21, 35]. However, inspection of shipping goods or port areas in the native country of *H*. *halys* to assess risk levels of overwintering *H*. *halys* to hitchhike to export goods or vessels has not been reported yet.

This study reports for the first time the detection of overwintering *H*. *halys* from a port of export in the Republic of Korea where a large volume of export goods including vehicles are loaded and shipped to international markets [36]. Four overwintering adults were found solitarily in Pyeongtaek port. However, no large aggregation of *H*. *halys* was detected in this study, different to reports from the invaded region such as the U.S. [16, 17]. In general, overwintering *H*. *halys* adults were found in gaps or crevices of structures in Pyeongtaek port known to share common features with favorable overwintering habitats demonstrated in previous studies [20, 31]. For examples, Inkley [17] demonstrated that a large number of aggregating *H*. *halys* were found overwintering in dry and tight conditions in attics. In this study, three overwintering adults were found from the gap between piled cardboard boxes, the gap between vinyl wraps and a bamboo frame, and a tight space under a plastic panel. Indeed, inspectors generally assigned higher likelihood evaluation scores for these structures. Evaluation scores of these overwintering habitats were ranked 6th and 16th out of 57 structures inspected; statistical analysis was not attempted between the likelihood score and the number of overwintering *H*. *halys* due to the overwhelming zero count in the data (Table 2). Interestingly, however, one individual was found somewhat unexpectedly from a crevice in the cement floor supporting a light tower in the port. The light tower was assigned with a relatively low score by inspectors as a potential overwintering site of *H*. *halys* because the tower was mainly made of steel with few tight gaps between components. In addition, the cement floor was not thought as a potential overwintering site because micro-climates near the ground are typically humid and subject to freezing. However the crevice from which *H*. *halys* was found seemed to have remained dry for a while before the survey was conducted, because the port was completely paved and designed for rapid outflow of running water. However, we cannot rule out the possibility that the crevice would become wet with precipitation that might cause mortality of overwintering individuals.

Results of this study indicate that the likelihood of *H*. *halys* to overwinter and form large aggregations in ports of export in the Republic of Korea is low. Indeed, only four overwintering *H*. *halys* were found from intensive visual inspection in ports of export over the two-winter season survey. This might result from a suite of reasons including the following. First, the overall size of overwintering *H*. *halys* populations is thought to be substantially smaller in the Republic of Korea than in some countries such as the U.S. from which serious nuisance problems of overwintering *H*. *halys* have been repeatedly reported [8, 17, 37]. For examples, citizen scientists, primarily from the Mid-Atlantic regions of the U.S., counted the number of *H*. *halys* present on the exterior of their homes during the autumn dispersal period [16]. Results of Hancock *et al*. [16] indicate that the mean peak counts of *H*. *halys* on the home exterior are in the range of ca. 150 to 550. To our knowledge, this high numbers of *H*. *halys* aggregation have not been reported in the Republic of Korea. Second, the urban landscapes in and near the ports of export would be expected to have a low density of *H*. *halys*, resulting in a low number of overwintering insects in human-made structures. Hancock *et al*. [16] reported that the number of *H*. *halys* observed on the home exterior was significantly lower in urban landscapes compared to rural areas. The two ports, Ulsan and Pyeongtaek, are located in industrial complexes adjacent to city districts. In addition, climate conditions, especially with regard to wind speed, may affect the tendency of *H*. *halys* to disperse into ports of export for overwintering [9, 38]. Given that *H*. *halys* is known to initiate its flight under calm or very mild wind conditions, frequent high-speed wind or wind gust in waterfront areas of ports would discourage active flight of *H*. *halys* in the areas [9].

Although it would not be quite practicable to pinpoint the origin of *H*. *halys* found overwintering in the port, *H*. *halys* might have flown from their feeding sites to the port of export for overwintering when weather conditions were favorable for dispersal flight. In laboratory conditions, *H*. *halys* was demonstrated to fly on average 2 km over 22 hours [9]. In this study, we monitored *H*. *halys* populations using pheromone traps in wooded and agricultural areas with varying distances from the ports. Our monitoring results suggest that *H*. *halys* populations were present in the vicinity of the two ports (e.g., 1.8 km away from Pyeongtaek port). Indeed, an on-going research study indicates that *H*. *halys* could be captured consistently from two wooded areas near both ports from April to November (unpublished data). These populations could serve as a source of overwintering individuals found in Pyeongtaek port. In addition to self-powered dispersal, it is also possible that export items had been infested with overwintering *H*. *halys* at other sites (e.g., manufacturing facility), and those individuals were transferred to port areas.

Identifying the origin of invasive species is fundamental to prevent invasion of alien species and establish quarantine levels appropriately between trading countries [21, 34]. Such information is also essential to design and implement sustainable management strategies such as classical biological control programs [39, 40]. In general, the origin of invasive species has been identified by tracking back routes of export goods from which the invasive species was found [6, 21, 34, 41]. For example, *Lymantria dispar asiatica* (Vnukovskij), formerly called the Asian gypsy moth, was found to infest vessels departing from the Russia Far East, and upon arrival in North America eggs hatched into larvae ballooning to vegetation surrounding port areas [23]. Recently, sequencing mitochondrial DNA has been used for tracking origins of *H*. *halys* [27, 42, 43]. In addition to a forensic-type tracking of *H*. *halys* origin, this study provides basic data to evaluate risk levels of *H*. *halys* to invade export goods or vessels at outbound borders of the native country of this species. Results of this study indicate that the likelihood of *H*. *halys* to form large overwintering aggregations in ports of export is relatively low in the Republic of Korea. Still, it is important to continue monitoring in the native country because previous studies indicate the occurrence of multiple invasions of this pest from Asia to new region [42]. This information may help quarantine authorities make more judicious and timely decisions on quarantine practice levels between trading countries.

## Supporting information

S1 Data (XLSX)
